# Impact of Hydrous Manganese and Ferric Oxides on the Behavior of Aqueous Rare Earth Elements (REE): Evidence from a Modeling Approach and Implication for the Sink of REE

**DOI:** 10.3390/ijerph15122837

**Published:** 2018-12-12

**Authors:** Haiyan Liu, Olivier Pourret, Huaming Guo, Raul E. Martinez, Lahcen Zouhri

**Affiliations:** 1 State Key Laboratory of Biogeology and Environmental Geology, China University of Geosciences, Beijing 100083, China; zwang@cugb.edu.cn (H.L.); hmguo@cugb.edu.cn (H.G.); 2 Guangdong Provincial Academy of Environmental Science, Guangzhou 510000, China; 3 UniLaSalle, AGHYLE, 60026 Beauvais CEDEX, France; lahcen.zouhri@unilasalle.fr; 4 Institut für Geo- und Ulweltnaturwissenschaften, Albert-Ludwigs Universität, 79104 Freiburg, Germany; rmartinez@bgc-jena.mpg.de; 5 Max-Planck-Research Group Paleobiogeochemistry, University of Bremen, 28359 Bremen, Germany

**Keywords:** rare earth elements, adsorption, hydrous manganese oxides, hydrous ferric oxides, surface complexation modeling, lanthanides

## Abstract

In this study, models were used for the first time to investigate the fate and transport of rare earth elements (REE) in the presence of hydrous manganese and ferric oxides in groundwaters from the coastal Bohai Bay (China). Results showed that REE sorption is strongly dependent on pH, as well as hydrous manganese and ferric oxide content. Higher proportions of REE were sorbed by hydrous manganese oxide as compared to hydrous ferric oxides, for example in the presence of neodymium. In this case, a mean 28% of this element was sorbed by hydrous manganese oxide, whereas an average 7% sorption was observed with hydrous ferric oxides. A contrasting REE sorption behavior was observed with hydrous manganese and ferric oxide for all investigated groundwaters. Specifically, REE bound to hydrous manganese oxides showed decreasing sorption patterns with increasing atomic number. The opposite trend was observed in the presence of hydrous ferric oxides. In addition, these results suggested that light REE (from La to Sm) rather than heavy REE (from Eu to Lu) are preferentially scavenged by hydrous manganese oxide. However, the heavy REE showed a greater affinity for hydrous ferric oxides compared to light REE. Therefore, both hydrous manganese and ferric oxide are important scavengers of REE. This study shows the implication of hydrous manganese and ferric oxide sorption for the sink of REE in groundwater.

## 1. Introduction

Rare earth elements (REE) concentrations in groundwaters vary depending on specific water–rock interactions, and the presence of dissolved or colloidal organic and inorganic species. Several studies have shown that unique REE signatures can be obtained, which are representative of the types of aquifer rock material through which the water is flowing [1,2,3,4,5,6]. The variable REE content of groundwaters, however, results from a number of different chemical parameters. It has been previously observed, for example, that a high REE content arises from slightly acidic conditions in waters with high CO_2_ concentrations. Under near-neutral conditions, the proportion of dissolved REE is low, with lowest REE levels found in alkaline groundwaters [7]. The physicochemical parameters of groundwaters, as well as rock mineral aquifer compositions, play therefore a fundamental role in the resulting REE concentrations and chemical speciation in the natural environment. As a result of this complexity, the chemical forms of the REE in groundwaters are for the most part controlled by the composition of aquifer minerals, pH, oxidation–reduction conditions, as well as, REE complexation to organic and inorganic ligands, and colloidal and particle matter transport [3,5,7].

In the ocean, hydrothermal vents have been suggested to be a net sink of REE due to their complexation with Fe and Mn oxides produced in the plume [8]. A similar mechanism involving complexation of the REE to Fe and Mn oxides has been proposed in groundwater [2,9,10]. The unique chemical properties of the REE allow them to form complexes with ubiquitous reactive solid surfaces, including those of organic matter and Fe and Mn oxides [11,12]. However, characterization of these REE forms along with a detailed quantification of their transport and fate in natural groundwater is still lacking.

Despite the advances in the understanding of REE surface complexation with Fe and Mn oxides [13,14] and the numerous studies on REE behavior in groundwater [2,3,5,6,9,15], the competition of the REE for surface complexation in the presence of Mn and Fe oxides reactive solids has not yet been fully quantified. Furthermore, the surface properties of Mn oxides are different from those of their Fe counterparts [16,17]. Both of these oxides, however, are ubiquitous in nature and control REE fractionation and mobility in groundwater. However, it is essential to quantitatively describe REE binding to Fe/Mn oxyhydroxides, in order to determine the extent to which REE groundwater patterns depend on fractionation of the REE by hydrous manganese oxides (HMO) and hydrous ferric oxides (HFO) and to better understand the fate and transport of the REEs in groundwaters. 

Therefore, the objectives of this study are: (i) to determine REE abundances and fractionation in submarine groundwaters and better understand the behavior of particle bound REE and their influence on seawater composition as a result of submarine groundwater discharge, and (ii) to determine the geochemical processes mobilizing and fractionating REE during transport (i.e., discriminate between the ability of HMO and HFO to partition the REE).

## 2. Materials and Methods 

### 2.1. Regional Hydrogeological Settings

**Figure 1:**
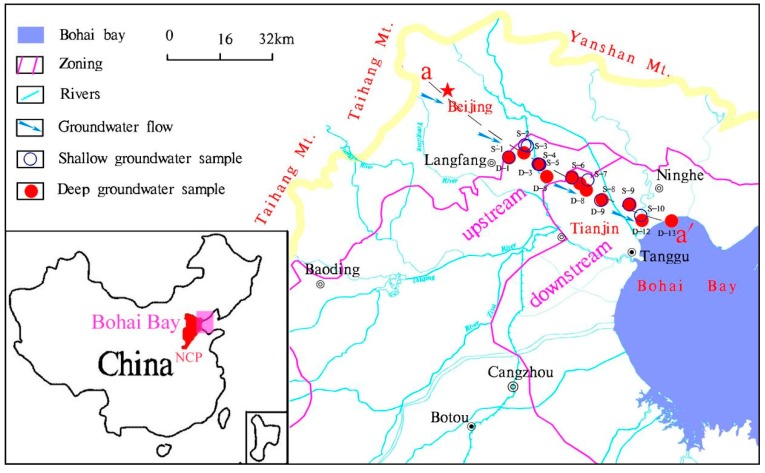
Study area and sampling locations.

The Bohai Bay is located in northeast region of China and is the second largest bay of the Bohai Sea. It covers one fifth of the total area of the Bohai basin and represents an important discharge zone of ground and surface waters. The Bohai bay is a typical semi-enclosed inner sea with an annual average temperature and precipitation of 11.9 °C and 660 mm, respectively [18]. The Bohai Bay slopes southward from an altitude of around 30 m above land surface (a.l.s) in the north to approximately around 2 m a.l.s in the south (Figure 1). The landform changes from alluvial-proluvial plain in the piedmont area to proluvial-marine plain at the coastal area. Three hydrogeological units from the piedmont zone to the littoral area correspond to: (1) the piedmont alluvial-proluvial plain, (2) the central alluvial-lacustrine plain, and (3) the eastern alluvial-littoral plain [19]. The present study focuses on the latter two. In this study, the central alluvial-lacustrine plain was ascribed to be upstream and the alluvial-littoral plain as being downstream, according to groundwater flow patterns and changing landform (Figure 1).

In this region groundwater occurs in Quaternary deposits composed of sandy gravel, medium-fine and fine sand [20] with a thickness of 30 to 50 m in the south, and up to 260 m in the north. The underlying basement mainly consists of metamorphic rocks of Archean to Proterozoic age. Aquifers can be classified according to their lithology, hydrodynamics and geologic age. Those with a burial depth of <200 m belong to shallow aquifers, whereas those at >200 m are considered deep aquifers [21]. Groundwater recharge occurs mainly by meteoric water via precipitation infiltration. A portion of the recharge arises from lateral and vertical leakage from deep aquifers. Groundwater discharge, however, is dominated by artificial pathways (e.g., pumping). Groundwater in the Bohai Bay mainly flows from the northwest to the southeast. However, extensive overexploitation has changed the natural groundwater flow patterns, which resulted in a serious seawater intrusion and the formation of a cone of depression induced by a continuous fall in groundwater level [22]. 

### 2.2. Groundwater Sampling

Groundwater samples were collected from electric-powered public wells used for drinking water supply or agricultural irrigation. Ten samples were collected from shallow aquifers and thirteen from deep aquifers. The samples were obtained along the profile a-a’ extending from Bohai seaside to the vicinity of Langfang, with a total distance of approximately 80 km (Figure 1). The depth of the investigated wells ranges from 7.3 m to 600 m below land surface. In order to ensure that the collected samples were representative of groundwater from the aquifer rather than the borehole, all wells were pumped until the pH, temperature, electrical conductivity (EC), and oxidation–reduction potential (ORP) remained stable. All sample vessels used in the field were pre-cleaned using acid-washed and deionized water in the laboratory and rinsed three times with extracted groundwater prior to sampling. All water samples were filtered with 0.45 μm cellulose acetate filters. The filtered groundwaters, were analyzed for major cations (Ca^2+^, Mg^2+^, K^+^, Na^+^), REE and other trace elements. The samples were stored in 100 mL high density polyethylene bottles and immediately acidified to pH < 2 by addition of 6 mol/L purified-HNO_3_. Groundwater samples for major anion analysis were collected without acidification. Groundwater samples for dissolved organic carbon (DOC) analysis were sampled in 30 mL amber glass bottles and immediately acidified with 1:9 (volume) H_2_SO_4_ to pH < 2.0. All samples were stored at 4 °C prior to analysis.

### 2.3. Chemical Analysis 

Physiochemical parameters including T, pH, EC and ORP were monitored in the field by using a HI 9828 portable multi-meter (HANNA, Woonsocket, RI, USA). Groundwater physicochemical conditions were maintained by letting it flow through an in-line flow cell with minimal atmospheric contact. The instrument was calibrated using standard solutions before use. Alkalinity was determined on-site with a Model 16900 digital titrator (HACH, Loveland, USA), standard purified H_2_SO_4_ (0.80 mol/L), and a bromocresol green-methyl red indicator. Concentrations of total Fe, Fe(II), nitrite, ammonium and sulfide were measured using a DR2800 portable UV/VIS spectrophotometer (HACH, Loveland, CO, USA), calibrated with corresponding standard curves. Total Fe and Fe(II) were determined using 1.10 FerroVer and phenanthroline methods, respectively [4,20]. The ORP values reported in this study have not been corrected to the standard hydrogen electrode (SHE), but instead can be used as relative values [4].

Groundwater major anion (e.g., Cl^−^, NO_3_^−^, and SO_4_^2−^) concentrations were analyzed using an ion chromatography system (ICS2000, Dionex, Thermo Fisher Scientific, Waltham, MA, USA), with a precision better than 3%. For major cations and trace elements analysis, Inductively Coupled Plasma Atomic Emission Spectrometry (ICP-AES: iCAP6300, Thermo Fisher Scientific, Waltham, MA, USA) and Inductively Coupled Plasma Mass Spectrometry ICP-MS; 7500C, Agilent Technologies, Santa Clara, CA, USA) were used, respectively, with analytic precisions better than 2%. Samples with elevated Mn and Fe concentrations were diluted appropriately to fit the standard curve during analysis. Validation of chemical data using the charge balance methods showed that all tested samples had a precision better than 5%.

Groundwater REE concentrations were quantified by ICP-MS (7500C, Agilent Technologies, Santa Clara, CA, USA). Sample treatment and chemical analyses followed the routine protocol described previously [4,20]. 

### 2.4. Surface Complexation Modeling

The speciation modeling for Mn and Fe oxides equilibrium calculations was achieved with the hydrogeochemical code PHREEQC version 3.3.9 [23] using the Nagra/PSI database [24]. The Nagra/PSI database was updated by the incorporation of well-accepted stability constants for the 14 naturally occurring REE at zero ionic strength and 25 °C. These constants were obtained from previous studies and account for inorganic aqueous complexes with: carbonate, hydroxyl, sulfate, chloride, and fluoride anions [10,25,26,27,28,29,30,31,32]. Specifically, precipitation of HMO and HFO was quantified from measured Mn and Fe in groundwater samples and the corresponding equilibrium constants from the literature [33]. The concentration of REE binding sites of the reactive solid surfaces was determined by the moles of HMO or HFO, and defined explicitly by the keyword data block “EQUILIBRIUM PHASES”. The specific surface area (SSA) was defined relative to the moles of HMO or HFO, in which the amount of specified binding sites changed as the SSA varied during batch-reaction simulation. Upon HMO or HFO formation, two types of oxide surface binding sites (=S^s^OH and =S^w^OH) were assumed to be available for REE complexation. For surface complexation modeling, both the surface-bound and diffuse layer species, were taken as the components of the system in the presence of manganese and iron oxyhydroxides. It must be noted that REE surface complexations to HMO and HFO were modeled separately for a given sample, because the present model is unable to consider both HMO and HFO simultaneously. This issue is a matter of ongoing investigation, in which a component additivity approach could be considered [34]. All the REE considered in the model were trivalent, because following the hypothesis of Bau [35], oxidation scavenging of Ce by metal oxides consists of three independent steps: (i) sorption of Ce(III) from solution, (ii) partial oxidation of Ce(III) to Ce(IV) on the iron or manganese oxide surface, (iii) partial desorption of Ce(IV) to solution.

## 3. Results

### 3.1. General Groundwater Chemistry

**Figure 2:**
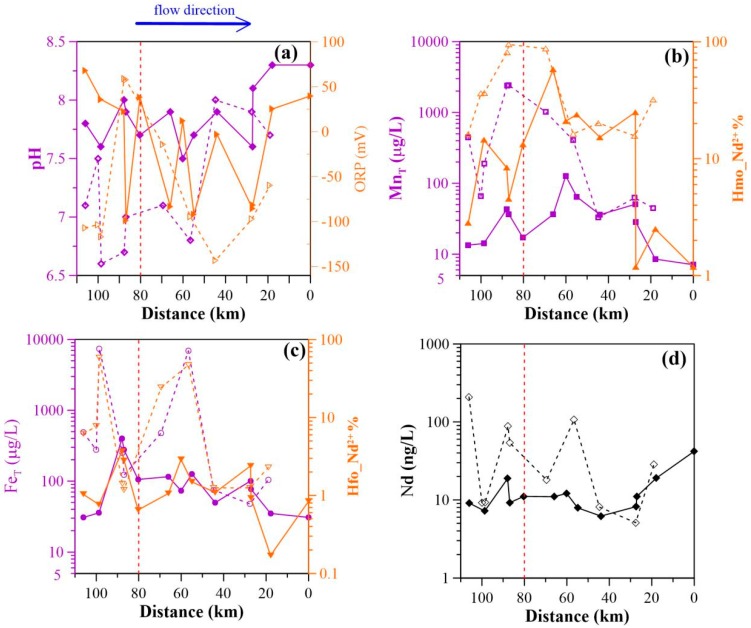
(**a**) Physicochemical parameters (pH, ORP) and (**b**) manganese concentration and proportion of Nd sorbed by hydrous manganese oxides (HMO), (**c**) iron concentration and proportion of Nd sorbed by hydrous ferric oxides (HFO) and (**d**) concentrations of Nd as a function of distance from the sampling location of sample D-13 (Bohai seaside) (The dash line denotes shallow groundwater, while the solid line indicates deep groundwater; pH, ORP, Fe, Mn, Nd parameters were determined by experiment; the amounts of Nd sorbed by HMO and HFO were determined by modeling).

The major components of all groundwater samples along with ancillary physicochemical parameters are presented in Appendix (Table A1). The pH of shallow groundwater ranged from 6.65 to 7.96, and that of deep groundwater from 7.52 to 8.33. Generally, the pH increased as the sampling locations approached the Bohai Sea (Figure 2a). Total dissolved solids (TDS) ranged from 329 to 9359 mg/L, and from 347 to 929 mg/L for shallow and deep groundwaters, respectively. Among the shallow groundwater samples, 5 had TDS greater than 1000 mg/L, while for deep groundwater values were below 700 mg/L. The high TDS groundwaters were mainly distributed in the upstream (Table A1). Na^+^ and HCO_3_^−^ were the major cation and anion species for all deep groundwaters, as well as for shallow groundwater samples downstream (except for S-6) (Figure 2). Upstream, different types of shallow groundwaters were observed with Cl^−^ being the dominant anion (e.g., S-1 to S-5 and S-7). 

The oxidation–reduction potential (ORP) ranged from −143 mV to 59 mV (mean −62 mV) in shallow groundwater, and from −99 mV to 68 mV (mean −16 mV) in deep groundwater, respectively, showing moderately reducing conditions. Generally, the ORP was higher upstream, compared to the downstream groundwater, although it was highly variable along the flow path (Figure 2a). Concentrations of NO_3_^−^ and SO_4_^2−^ had average values of 93 mg/L and 359 mg/L in shallow groundwater, respectively. These were higher than those of deep groundwater, with corresponding values of 5 mg/L and 34 mg/L. Relatively high NH_4_^+^ and ΣS-II concentrations were observed in shallow groundwater, with ranges varying from <0.01 mg/L and 2.5 mg/L, and from <1.0 μg/L and 76 μg/L, respectively. In comparison with shallow groundwater, NH_4_^+^ concentrations were relatively low (generally <0.1 mg/L) in deep groundwater; while S^2−^ concentrations ranged from <1.0 μg/L to 49 μg/L.

Total dissolved iron (Fe_T_) concentrations ranged from (Figure 2b) 48 µg/L to 7382 μg/L, and from 31 µg/L to 397 µg/L with an average value of 1612 µg/L and 128 µg/L for shallow and deep groundwater samples, respectively (Figure 2b). Iron (II) was present in a significant amount, ranging from 10 µg/L to 2200 µg/L (with an average value 364 µg/L) and from 10 µg/L to 350 µg/L (55 µg/L) in shallow and deep groundwater samples, respectively. Along the flow path, shallow groundwater Fe_T_ concentrations fluctuated with a relatively high average value (>250 µg/L) in the upstream before progressively decreasing for the remaining path sampled (downstream). Deep groundwater Fe_T_ concentrations generally decreased with groundwater flow (Figure 2b).

Total dissolved manganese (Mn_T_) concentrations were lower than Fe_T_ concentrations. Shallow groundwater had Mn_T_ concentrations extending from 33 µg/L to 2429 μg/L with an average value of 709 µg/L. In groundwater from deep aquifers, Mn_T_ concentrations ranged from 7 µg/L to 127 μg/L (average 42 µg/L). A decreasing trend for Mn_T_ concentrations was observed in shallow groundwater as a function of distance from the Bohai seaside (Figure 2c). Total Mn concentrations of deep groundwater samples initially showed a small increase along the sampling path until the location of the sample D-7 (the highest value). From this point on, the Mn_T_ concentrations gradually diminished to the end of the flow path (Figure 2c). 

Overall, shallow groundwaters had higher concentrations of Mn_T_ and Fe_T_ than deep groundwater samples. Upstream samples had higher Mn_T_ and Fe_T_ concentrations with respect to those from the downstream. All groundwater samples showed low DOC concentrations (ranging between 1.5 mg/L and 4.5 mg/L (mean 2.2 mg/L) in shallow groundwater, and between 0.8 and 2.9 (mean 2.2 mg/L) in deep groundwater) (see Table A1).

### 3.2. REE Concentrations and Normalized Patterns

Rare earth element concentrations and fractionation indices are shown in the Supplementary Information (Table A2). Total REE (∑REE) concentrations of shallow groundwater ranged from 51 ng/L to 1141 ng/L with an average value of 312 ng/L, whereas ∑REE concentrations of deep groundwater ranged from 51 ng/L to 228 ng/L (average 96 ng/L). Low pH shallow groundwaters containing high REE levels were mainly distributed upstream (Table A2). Contrary to the shallow groundwater, ∑REE concentrations of deep groundwater do not vary substantially. Higher ∑REE were determined in samples distributed near the end of the sampled path (i.e., D-12 and D-13) (Table A2).

**Figure 3:**
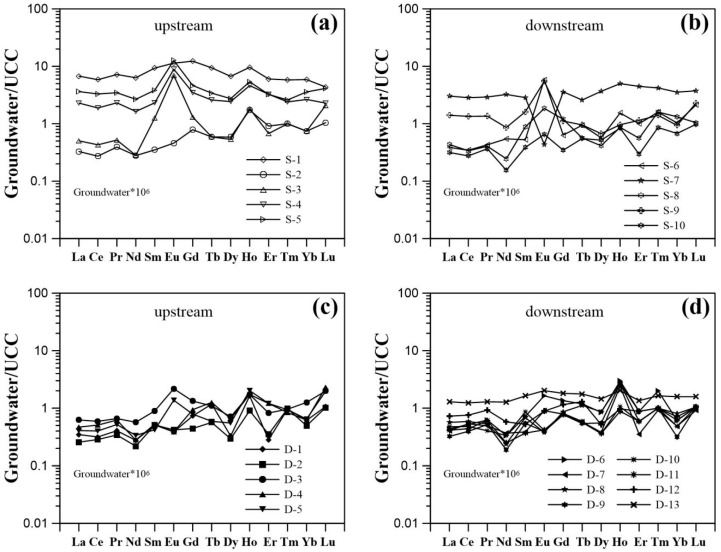
Groundwater upper continental crust (UCC)-normalized REE patterns of (**a**) upstream and (**b**) downstream shallow groundwater samples; and (**c**) upstream and (**d**) downstream deep groundwater samples (UCC values are from McLennan [36]).

Rare earth element concentrations were normalized with the average REE composition of the upper continental crust (UCC) [36] (Figure 3). Two fractionation indices ((Gd/Nd)_UCC_ and (Yb/Nd)_UCC_) were employed as a measure of groundwater REE fractionation with respect to UCC. The resulting REE patterns were characterized by enrichment of middle REE (MREE) and heavy REE (HREE) relative to light REE (LREE) (except for S-1), as suggested by (Yb/Nd)_UCC_ and (Gd/Nd)_UCC_ ratios. These values ranged between 0.9 and 5.4 (average 2.3), and between 1.1 and 4.9 (average 2.4) for shallow groundwater, respectively; those for deep groundwater ranged between 1.2 and 3.4 (average 2.0), and between 1.4 and 4.1 (average 2.7), respectively. All of the UCC-normalized REE patterns for shallow groundwater samples had negative Ce anomaly (Ce/Ce* = [Ce]_UCC_/([La]_UCC_ × [Pr]_UCC_)^0.5^) ranging between 0.76 and 0.97 (average 0.86). Generally, a negative Ce anomaly (ranging between 0.84 and 1.20) has been observed for deep groundwater samples with an average value of 0.97. Groundwater samples from upstream generally showed smaller values for the negative Ce anomaly with respect to those from downstream. Eu anomalies (Eu/Eu* = [Eu]_UCC_/([Sm]_UCC_ × [Gd]_UCC_)^0.5^) ranging from 0.1 to 10.1 (average 3.14) and from 0.5 to 2.4 (average 1.1) were observed in shallow and deep groundwater samples, respectively. Positive Eu anomalies occurring upstream may be attributed to water–rock interaction involving Eu-bearing minerals (e.g., feldspar) [20].

### 3.3. Surface Complexation Modeling Results

**Figure 4:**
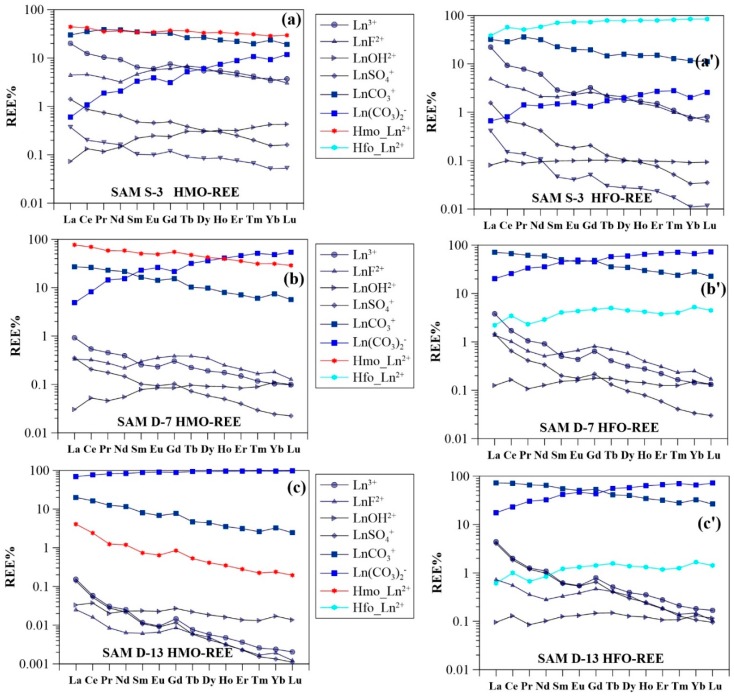
Results of REE speciation modeling (Ln represents any of the REE). (**a**) sample S-3 considering HMO; (**a’**) sample S-3 considering HFO; (**b**) sample D-7 considering HMO; (**b’**) sample D-7 considering HFO; (**c**) sample D-13 considering HMO; (**c’**) sample D-13 considering HFO.

Speciation modeling of REE surface complexation to HMO and HFO predicted that a significant amount of REE was sorbed by HMO at high Mn concentrations (≥10.0 μg/L) (Figure 4a–c), and that carbonate-REE species were dominant at lower dissolved Mn concentrations (<10.0 μg/L) (Figure 4). The fraction of the REE (i.e., Nd) sorbed by HMO averaged 44% for shallow groundwater, and 14% for deep groundwater. Trends for Nd sorbed by HMO generally increased in the region upstream and decreased progressively in the down-gradient part of the sampled path for both shallow and deep groundwaters (Figure 2b). A significant fraction of the REE, in particular for the LREE, was suggested to be present as dissolved Ln^3+^ species in upstream shallow groundwaters, with a low pH and high Mn content. Sample S-3 had a pH of 6.55 and a total Mn concentration of 199 μg/L. Using these parameters, the resulting fractions of La^3+^, La sorbed by HMO, and carbonate species (i.e., LaCO_3_^+^ and La(CO_3_)_2_^−^) were calculated to be 20%, 44%, 30% and <1%, respectively. In the presence of HMO, the fractions of Lu^3+^, Lu sorbed by HMO, and carbonate species (i.e., LuCO_3_^+^ and Lu(CO_3_)_2_^−^) were determined to be 4%, 30%, 19% and 12% (Figure 4a). The HFO scavenging model predicted a lower concentration of the REE bound by HFO, as compared to HMO. Only in groundwaters with high Fe concentrations (i.e., S-3 and S-7, having Fe concentrations of 7382 μg/L and 6912 μg/L, respectively), the bulk REE were predicted to sorb mostly on the Fe oxides (i.e., Nd sorbed by HFO were 58% and 47% for S-3 and S-7, respectively). The majority of the groundwater REE were modeled to occur as carbonate complexes (Figure 4a). In general, HFO complexes accounted for <1% to 58% with an average value of 15% for Nd in shallow groundwater. For deep groundwater these values were below 4% for all samples. Along the length of studied flow path, HFO complexes generally decreased from upstream to downstream. This was best observed in deep groundwater (Figure 2c).

## 4. Discussion

### 4.1. Mobility of REE in Groundwater

**Figure 5:**
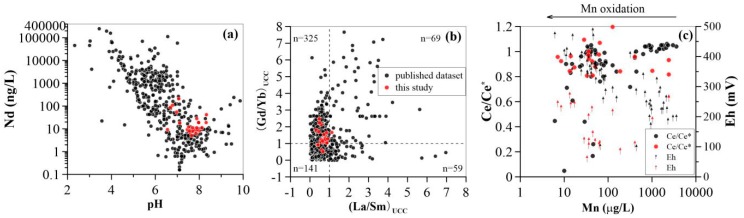
Comparisons of the data set in present study with those from the literature reviewed by Noack et al. [27]. (**a**): Nd concentrations vs pH; (**b**): (La/Sm)_UCC_ vs (Gd/Yb)_UCC_; (**c**): Mn concentrations vs. Ce anomaly and Eh. Data sources displayed in panel c are from [2,3,5] (black points and arrows) and the present study (red points and arrows).

Groundwater REE concentrations were highly variable along the groundwater flow path. A decreasing trend for tracer Nd concentrations was observed in shallow aquifers, whereas in deep groundwaters these concentrations were constant, except for samples D-12 and D-13. These sampling locations were placed near the end of flow path, with Nd concentrations of 19.0 ng/L and 41.1 ng/L, respectively (Figure 2d). This is attributable to changing pH, where lower-pH shallow groundwaters upstream (see Figure 2d)) contained higher Nd concentrations, than those with higher-pH values (Figure 5a). The observed trend of Nd concentrations as a function of pH is consistent with that of literature groundwater data sets [7] (Figure 5a). Consequently, the variations in groundwater REE concentrations result from pH change in shallow aquifers upstream. While other processes such as sorption and groundwater flow downstream in deep aquifers, may affect the transport and sink of groundwater REE in deep aquifers.

Previous studies showed that REE groundwater concentrations originated from reactions involving relevant minerals in aquifer sediment/rocks with newly recharged water (i.e., meteoric water) [37,38]. This results in groundwater REE patterns which resemble those of the aquifer rocks, in regions proximal to recharge zones [6,15]. Hence, the groundwater REE geochemistry (i.e., concentration and normalized patterns) arose from water–rock interactions [6] and changes in solution chemistry [2], as well as from the residence time within the aquifer through which the groundwater flows [39]. 

Geochemical modeling showed that groundwater samples were all undersaturated with respect to amorphous silica (SiO_2_(a)). Calcite and dolomite were generally found to be undersaturated in shallow groundwater samples from upstream. Two deep groundwater upstream samples (D-1 and D-2) were undersaturated with respect to calcite, and dolomite as well as aragonite. In consequence, chemical weathering and/or dissolution of calcite and dolomite were favorable in the upstream and silicate dissolution occurred along the entire flow path. This weathering process is enhanced by the lower pH resulting from the decay of organics under anaerobic conditions. This was reflected by the coexistence of NO_3_^−^ and NH_4_^+^ and SO_4_^2−^ and ΣS-II. The REE, as well as major and trace elements were mobilized from the host rock under acidic conditions and oxidized water percolation [38]. This is consistent with the high levels of REE concentrations observed, in shallow groundwater upstream (i.e., samples S-1, S-4 and S-5). 

REE fractionation patterns were a result of REE sorbed onto the surface of newly formed secondary minerals, such as Fe and Mn oxides/oxyhydroxides (Figure 2b,c). Since the absolute concentrations of LREE are higher than those of MREE and HREE in groundwater, preferential scavenging of LREE over MREE and HREE led to enrichment of MREE and HREE in solution. Therefore, in these weakly acidic groundwater samples, the aqueous REE speciation was mainly driven by the carbonate complexes (i.e., LnCO_3_^+^), in particular for the LREE (Figure 4a). 

Downward migration of groundwater in shallow aquifers and infiltration into deeper aquifers, gave rise to moderately alkaline groundwater. In these deep and shallow groundwater samples downstream, aqueous REE mainly formed carbonate complexes (Ln(CO_3_)_2_^−^). The lower proportion of sorbed REE (i.e., Nd) in deep groundwater samples (Figure 4) and their decreasing trends (Figure 2b,c) further evidenced the weaker sorptive effect of Fe and Mn oxides/oxyhydroxides on groundwater REE. Thus, the HREE and MREE were preferentially mobilized with respect to the LREE as a result of the preferential stabilization of the LREE by carbonate complexation reactions. This led to HREE and MREE enrichment in deep and shallow groundwaters downstream, but to lower overall dissolved REE concentrations (Figure 2d). This mechanism may reflect the importance of Fe and Mn oxide colloids on the sorption and transport of REE and could further explain the lower REE concentrations in deep groundwater, as compared to shallow groundwater (Figure 2d). The higher REE concentrations in deep groundwater near the end of the flow paths (i.e., sample D-12 and D-13) can result from the mixing with shallow groundwater, as the water type in shallow groundwater (i.e., sample S-10) downstream was the same as that of the deep groundwater (i.e., samples D-12 and D-13). 

In this study, neutral and weakly alkaline pH groundwater (Table A1) showed MREE- and HREE-enriched normalized patterns (see (Gd/Nd)_UCC_ and (Yb/Nd)_UCC_ ratios in Table A2). Indeed, further exploration of the extent of REE fractionation with (La/Sm)_UCC_ and (Gd/Yb)_UCC_ ratios indicated that the samples showed a general trend of REE fractionation in groundwater (Figure 5b). All samples (except for S-7) had (La/Sm)_UCC_ ratio <1, and approximately 80% of samples had (Gd/Yb)_UCC_ ratio >1. This trends closely resembled those observed in the dataset in Figure 5b (594 groundwater samples reviewed by Noack et al. [7]). 

In consequence, groundwater MREE- and HREE-enriched fractionation patterns were controlled by competition between REE solution complexation and surface complexation. This process was primarily driven by pH. 

### 4.2. Impact of Manganese

Manganese oxides/oxyhydroxides (i.e., MnO_2_) are important dominant scavengers for the REE in the natural environment [12,40], due to their low pH of zero point charge (pH_pzc_) (<4.0) [41]. Modeling of the results of aqueous REE scavenging by Mn oxide showed a decreasing downward trend for the proportions of the bound REE as a function of increasing atomic number (Figure 4). This property may be independent of the pH, Mn concentration, and ionic strength, since consistent results were obtained from the modeling of different groundwaters collected along a single flow path (Compare Figure 4a–c). In this respect, Mn oxide shows a preferential scavenging of LREE relative to HREE and MREE. This effect results in REE signatures showing enrichment of HREE and MREE with respect to LREE in solution as shown in Figure 3 and as indicated by (Gd/Nd)_UCC_ and (Yb/Nd)_UCC_ ratios (Table A1). It should be noted that the HREE-enriched patterns, as well as the negative Ce anomaly of ocean waters are attributed to oxidative scavenging by Mn oxides/oxyhydroxides [42,43].

**Figure 6:**
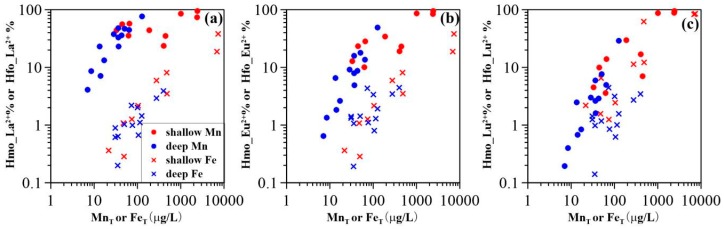
Proportion of (**a**) La, (**b**) Eu, and (**c**) Lu sorbed by HMO and HFO as a function of total Mn concentrations (Mn_T_) and total Fe concentrations (Fe_T_) (The sorbed La, Eu, and Lu were determined by modeling; Mn_T_ and Fe_T_ concentrations were determined by experiments).

**Figure 7:**
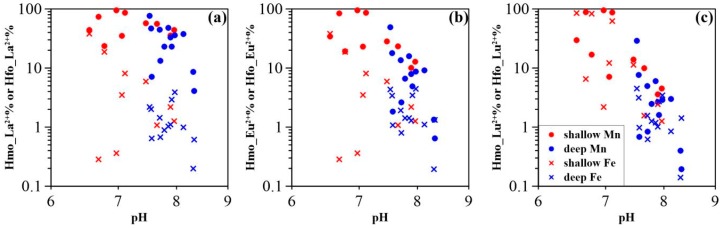
Proportion of (**a**) La, (**b**) Eu, and (**c**) Lu sorbed by HMO and HFO as a function pH (The sorbed La, Eu, and Lu were determined by modeling).

**Figure 8:**
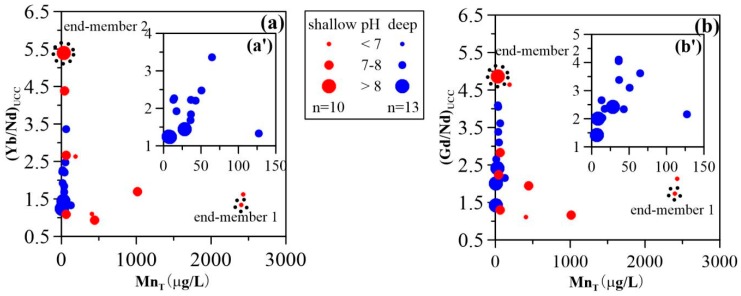
(**a**) (Yb/Nd)_UCC_ ratio (**b**) (Gd/Nd)_UCC_ ratio as a function of total Mn concentrations (Mn_T_) for shallow and deep groundwater (UCC values are from McLennan [36]) (REE, Mn_T_ and Fe_T_ concentrations were determined by experiments).

REE sorption behavior was strongly dependent on pH and reactive Mn and Fe oxides/oxyhydroxide concentrations [44,45]. The REE fractional sorption (illustrated by La, Eu, Lu) was found to be significantly affected by Mn concentrations (Figure 6); and groundwater pH values (Figure 7). This suggests that Mn oxides are the cause of discrepant REE signatures found in the sampled paths (i.e., upstream and downstream) as well as in shallow and deep aquifers. This can be inferred from the results since Mn concentrations were higher upstream than downstream (Figure 2b,c), and the pH values of upstream groundwater were generally lower than those downstream (Figure 2a). Furthermore, lower groundwater Mn concentrations were observed in deep aquifers with lower pH values, as compared to shallow aquifers. Therefore, two end-members could be established with respect to the influence of Mn oxides on REE fractionation, namely (i) the varying pH and (ii) the Mn concentrations along groundwater flow path. As shown by dissolved Mn and (Yb/Nd)_UCC_ and (Gd/Nd)_UCC_ ratios in Figure 8a,b, one end-member with high Mn concentrations and lower (Yb/Nd)_UCC_ and (Gd/Nd)_UCC_ ratios is plotted in the lower right corner (EM1), while the other corresponding to low Mn concentrations and high (Yb/Nd)_UCC_ and (Gd/Nd)_UCC_ ratios (EM2) is shown in the upper left of Figure 8. The upstream shallow groundwater contained high dissolved Mn (i.e., the highest of 2430 µg/L) (Table A1). The Mn oxides colloids, however, were less prevalent at these low-pH conditions (i.e., lowest pH of 6.6). Although the model revealed a higher proportion of sorbed REE in the upper stream groundwater samples, REE sorption onto Mn oxides might be constrained by formation of carbonate, hydroxide, or organic complexes (not considered in the model). Groundwater REE were thus fractionated to a lesser extent due to the weaker sorption to Mn oxides. This is supported by values close to 1 for both (Yb/Nd)_UCC_ and (Gd/Nd)_UCC_ ratios, and by the higher concentrations REE in shallow groundwater (Figure 2d). This mechanism contributed to the ratios observed for end-member 1 in Figure 8. In shallow aquifers, Mn oxides were suggested to more strongly bind the REE due to the enhanced deprotonation of surface reactive binding sites as a function of higher pH [46]. This would lead to highly fractionated REE patterns (as recorded in end-member 2) and to lower REE concentrations, as observed in downstream shallow groundwaters (Figure 2d). As shown in Figure 8, (Yb/Nd)_UCC_ and (Gd/Nd)_UCC_ ratios in deep groundwater were for the most part positively correlated with Mn concentrations (Figure 6a,b). This indicated that Mn oxides REE sorption was mainly constrained by pH at low Mn concentrations (i.e., generally <50 μg/L), as deep groundwater pH values were more alkaline than those of shallow groundwater and were less variable along the sampling path (Figure 2a). 

### 4.3. Impact of Iron

In strong contrast to the Mn-oxide scavenging model, results of aqueous REE sorption by Fe oxide showed REE patterns with a concave-upward shape and a Yb_sorb_%/Nd_sorb_% ratio ranging between 1.3 and 8.2 and 1.1 and 2.0 in shallow and deep groundwater samples, respectively (Figure 4). These characteristics suggested that Fe oxide had a stronger affinity for HREE and MREE than for the LREE. However, this observation contradicts field and experimental results from the marine environment, where the LREE were preferentially accumulated in the solid Fe oxides/oxyhydroxides (i.e., FeOOH) [45]. 

For the majority of groundwater samples, aqueous REE were sequestered to a lesser extent by Fe oxide. This may be due to the higher pH_pzc_ of Fe oxide (i.e., 8.5 to 9.3) [41]. Under the physicochemical groundwater conditions in this study, the Fe oxide would be neutrally or weakly positively charged [40] disfavoring the sorption of REE. This effect may account for the lower fraction of REE sorbed on groundwater Fe oxide (Figure 7). Samples S-3 and S-7, however, with high Fe concentrations showed a higher proportion of the REE bound to Fe oxide, despite their low pH values (i.e., 6.6 and 6.8, respectively). This result suggested that Fe concentrations played an important role in the formation of Fe oxide and the subsequent sorption of groundwater REE. Indeed, a positively correlation between groundwater Fe and sorbed REE (i.e., La, Eu and Lu) was observed (Figure 6). In addition, REE sorption on the neutral Fe oxide surface may occur via proton exchange [47], which could explain the measurable REE sorption on Fe oxide at pH < 4 as shown previously [14,35]. Consequently, the decrease in proportions of REE sorbed by HFO along the groundwater flow path resulted from the decreasing Fe groundwater concentrations and the increasing pH values (Figure 2c). 

Another notable feature of Fe-oxide scavenging modeling results was that Ce was preferentially sorbed by Fe oxide with respect to La and Pr (Figure 4). The calculated Ce anomaly (Ce/Ce* = %Ce_sorb_/(%La_sorb_ × %Pr_sorb_)^0.5^) for Fe oxide sorbed REE patterns ranged between 1.3 and 1.8 in shallow groundwater, and had a value of approximately 1.6 in deep groundwater. The lowest Ce/Ce* was observed in groundwater sample S-3 (pH 6.5), taken from upstream shallow aquifers. This demonstrates that the stronger affinity of Fe oxide for Ce over La and Pr was evident under low pH conditions. Previous modeling studies showed that the Ce sorption edge for Fe oxide was below pH 5, and lower than that of La [30]. This mechanism is, for the most part, responsible for the lower negative Ce anomalies occurring in shallow groundwater (i.e., especially upstream) (Table A2), when compared to the deep groundwater condition. It is worth noting that in low-temperature aquatic systems, Ce is the only REE that can occur in a stable tetravalent state (i.e., as Ce(IV)). Oxidation scavenging of Ce(III) from aqueous solution onto oxide surfaces is recognized as the fundamental mechanism for the decoupling of Ce from its neighboring La and Pr in solution [8,48]. 

### 4.4. Comparison of HMO Scavenging Model and HFO Scavenging Model

**Figure 9:**
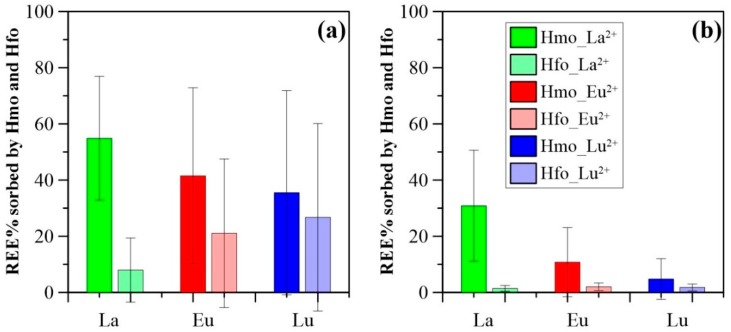
Comparison of sorbed La, Eu, and Lu by HMO and HFO for (**a**): shallow groundwater and (**b**): deep groundwater (The sorbed La, Eu, and Lu were determined by modeling).

From a viewpoint of surface complexation modeling, both Mn and Fe oxides were able to scavenge aqueous REE within specific physicochemical conditions (i.e., Mn and Fe oxide contents, and groundwater pH). However, the major difference between Mn and Fe oxides was the contrasting REE sorption patterns. REE sorption modeling with Mn oxide showed REE complexation in the order LREE > MREE > HREE, whereas Fe oxide, preferentially sorbed HREE > MREE > LREE. Since log Kd patterns showed little fractionation for experiments with the same REE concentrations. REE fractionation patterns in natural environment could be attributed to competitive sorption resulting from the high heterogeneity in absolute REE concentrations (LREE > MREE > HREE) (mentioned above). Another important difference between these two modeling results was the higher amount of REE sorbed on Mn oxide, as compared to Fe oxide for a given sample (Figure 9). The mean sorbed La concentration on Mn oxide was more than seven times higher than that with Fe oxide in shallow groundwater. Similarly, mean proportions of Eu sorbed by HMO were twice those of Lu sorbed by HMO, and 9 times higher than Lu sorbed by HFO (Figure 9a). For deep groundwater, however, all REE sorbed by HFO proportions were below 4 (Figure 9b). These results strongly suggest that Mn oxide exerts a stronger influence on groundwater REE, in comparison to Fe oxide. The MREE and HREE enrichment observed in groundwaters in this study were thus more likely attributable to the presence of Mn oxide, as mentioned earlier. 

Fractional sorption REE patterns on Fe oxide showed a positive Ce anomaly (stated above), which, has not been observed in the Mn-oxide scavenging model. This is because the redox properties of Ce(III) were not taken into account for REE speciation calculations. Recently, the aqueous chemistry of the Ce(IV) species has been investigated using actinide analogues [49]. Cerium can be significantly sequestered by Fe and Mn oxide-containing particles/colloids (i.e., ferromanganese nodules) due to its preferential scavenging by Mn and Fe oxides from solution with respect to La and Pr [16]. In addition, Nakada et al. [50] showed that Ce sorbed on ferrihydrite might not be oxidized in the Ce/ferrihydrite system as evidenced by XANES analysis and by the observed thermodynamic data [50]. In contrast, Mn oxide showed a stronger oxidizing potential for Ce(III) as compared to Fe oxide due to Mn oxide acting as a catalyst during Ce(III) oxidation [51]. It has been widely accepted that bound REE on Mn oxide frequently show positive Ce anomaly patterns resulting from oxidizing processes. Hence, role of Mn oxide in sequestration and accumulation of Ce is presumably coupled to Mn oxide content and ambient physicochemical settings (i.e., redox potential). Indeed, the observed negative Ce anomalies in this study were distributed as function of Mn concentrations, showing a clear resemblance to classical datasets [2,3,5] (Figure 5c). No significant changes in the size of Ce anomalies (shown above) in this study were caused by lower Eh values (Table A2). These values were comparable to those reported in literature, where approximately 90% of the samples’ Ce/Ce* values occurred between 0.8 and 1.1, despite a wide range in Eh (from 150 mV to 500 mV).

In consequence, although the redox properties of Ce(III) were not considered for the modeling the REE scavenging by Fe or Mn oxides in this study, the modeling approach yielded a systematic comparison of the REE sorption behavior on Mn and Fe oxides from a quantitative perspective. Moreover, it could motivate an extrapolation of the proposed data set to natural systems where manganese and/or iron oxyhydroxides prevail, unless other potential complexing agents (e.g., organics) are present and could therefore compete for REE sorption.

## 5. Conclusions

The results of our study showed that REE sorption exhibits a strong dependence on pH, as well as on Mn and Fe oxide content. Higher proportions of REE are sorbed by Mn oxide as compared to Fe oxide. This was well illustrated by the Nd sorbed by Mn, with respect to Fe oxide. As such, Nd sorbed by HMO ranged from 1% to 95% whereas Nd sorbed by HFO range from <1% to 58%. A contrasting REE sorption behavior was observed on Mn and Fe oxides for all investigated groundwaters. REE bound to Mn oxide showed decreasing sorption trends with increasing atomic number, in contrast to the Fe oxide condition. This suggests that LREE are preferentially scavenged by Mn oxide. Moreover, HREE show a greater affinity for Fe oxide compared to LREE. Therefore, both Mn and Fe oxides are important scavengers of the REE, showing the important role of Mn and Fe oxides for the sink of REE in groundwater. 

## Appendix A

The major components of all groundwater samples along with ancillary physicochemical parameters and elemental concentrations are presented in Appendix (Table A1 and Table A2).

**Table A1:** Physicochemical parameters (including pH, Eh, EC, TOC, major ions and total dissolved solid (TDS) of all groundwater samples.

Location		Sample No.	Distance (km)	Depth (m)	Eh (mV)	pH	Conductivity (μs/cm)	TDS (mg/L)	F^−^ (mg/L)	Cl^−^ (mg/L)	NO_3_^−^ (mg/L)	SO_4_^2−^ (mg/L)	HCO_3_^−^ (mg/L)	K^+^ (mg/L)	Na^+^ (mg/L)	Ca^2+^ (mg/L)	Mg^2+^ (mg/L)	Fe_T_ (μg/L)	Mn_T_ (μg/L)	TOC (mg/L)
Shallow	Upstream	S-1	81	12	96	7.1	4374	3260	5.2	977	127	557	783	1.7	751	183	268	485	444	2.2
		S-2	83	150	100	7.5	1094	850	2.1	43	15	97	648	0.8	310	31	26	276	65	2.7
		S-3	84	24	86	6.6	1452	968	1.7	200	4	5	749	2.2	118	194	68	7382	189	2.2
		S-4	96	7.3	261	7.0	3179	2576	3.7	801	138	311	677	2.9	486	356	139	122	2430	2.2
		S-5	97	6	262	6.7	3953	3343	2.0	961	414	454	703	0.3	554	437	169	249	2401	2.5
		S-6	112	150	189	7.1	7398	6250	12.2	2605	90	589	840	1.9	2009	226	296	476	1015	4.6
	Downstream	S-7	128	50	109	6.8	10050	9359	8.6	3036	134	1511	1611	13.8	3008	283	522	6912	407	1.5
		S-8	140	175	60	8.0	511	418	2.7	31	9	22	291	0.4	182	9	2	75	33	1.5
		S-9	170	170	144	7.7	483	392	0.7	45	3	23	279	0.5	151	17	3	48	45	1.6
		S-10	179	160	106	7.9	569	460	0.8	75	2	21	287	0.5	185	22	5	104	62	1.8
Deep	Upstream	D-1	75	320	271	7.8	596	439	2.1	46	8	71	317	0.5	178	13	3	31	13	0.9
		D-2	72	303	239	7.6	955	528	1.3	119	0	101	330	0.3	114	11	6	36	14	1.8
		D-3	66	390	225	8.0	783	606	1.1	68	7	29	405	0.8	254	20	7	397	43	3.0
		D-4	65	330	104	7.9	752	563	1.0	57	0	27	405	0.6	235	15	6	276	37	3.0
		D-5	62	400	241	7.7	989	621	1.4	55	4	5	573	0.6	221	10	4	106	17	2.3
	Downstream	D-6	56	375	120	7.9	707	551	3.7	33	3	21	429	0.6	246	11	3	115	36	2.8
		D-7	54	400	215	7.5	1280	929	1.1	224	4	33	537	0.7	326	46	24	73	127	2.9
		D-8	50	260	111	7.7	617	533	3.9	34	11	12	462	0.7	208	19	6	126	65	2.4
		D-9	42	235	200	7.9	510	396	2.2	39	6	27	258	0.4	163	11	2	50	36	1.3
		D-10	22	300	121	8.1	427	347	1.1	20	6	10	311	0.4	127	11	3	77	28	2.4
		D-11	21	240	118	7.6	493	414	1.8	39	2	16	323	1.2	169	14	9	101	51	2.1
		D-12	6	500	228	8.3	694	548	3.2	43	3	19	359	0.5	265	6	1	35	9	2.8
		D-13	0	600	243	8.3	585	506	1.3	37	7	72	258	0.4	228	6	1	31	7	2.2

**Table A2:** REE concentrations (ng/L) and fractionation parameters (Eu/Eu*, Ce/Ce*, (Gd/Nd)_UCC_, (Yb/Nd)_UCC_) of groundwater samples.

Location		Sample No.	La	Ce	Pr	Nd	Sm	Eu	Gd	Tb	Dy	Ho	Er	Tm	Yb	Lu	∑REE	Eu/Eu*	Ce/Ce*	(Gd/Nd)_ucc_	(Yb/Nd)_ucc_
Shallow	Upstream	S-1	216.1	426.9	56.9	208.9	53.2	14.1	63.9	8.0	38.8	10.0	20.5	2.9	18.2	2.1	1141	1.1	0.8	1.9	0.9
		S-2	10.5	19.9	3.1	9.2	2.0	0.6	4.1	0.5	3.4	1.8	3.1	0.5	2.3	0.5	61	0.9	0.8	2.8	2.7
		S-3	16.1	31.4	4.1	9.3	7.2	8.6	6.8	0.5	3.1	1.9	2.3	0.5	2.3	1.0	95	5.4	0.8	4.6	2.6
		S-4	73.3	137.4	18.3	54.0	13.2	11.1	18.1	2.2	14.1	4.8	11.1	1.2	8.2	1.1	368	3.1	0.8	2.1	1.6
		S-5	116.2	241.0	27.4	88.0	21.9	15.7	24.1	2.9	15.9	5.6	11.0	1.3	11.1	2.0	584	3.0	0.9	1.7	1.3
		S-6	12.4	25.2	3.4	18.0	3.0	7.2	3.3	0.8	3.3	1.6	3.4	0.8	3.2	1.0	87	10.1	0.8	1.2	1.7
	Downstream	S-7	96.9	208.0	23.1	106.8	16.3	0.5	18.7	2.2	21.3	5.2	15.2	2.1	11.0	1.8	529	0.1	1.0	1.1	1.1
		S-8	13.9	24.7	3.2	8.1	5.0	2.3	6.2	0.5	2.4	0.9	1.9	0.8	4.1	0.5	74	1.8	0.8	4.9	5.4
		S-9	10.1	20.1	2.9	5.1	2.2	0.8	1.8	0.5	3.0	0.9	1.0	0.4	2.1	0.5	51	1.8	0.8	2.2	4.4
		S-10	44.9	98.1	10.7	28.4	9.2	6.8	5.8	0.8	3.9	1.0	3.9	0.7	2.9	1.1	218	4.1	1.0	1.3	1.1
Deep	Upstream	D-1	11.1	23.1	3.2	9.1	2.9	0.5	3.8	1.0	1.9	1.7	1.0	0.5	1.9	0.5	62	0.6	0.8	2.7	2.2
		D-2	8.2	20.8	2.7	7.2	2.9	0.5	2.3	0.5	1.7	0.9	1.2	0.5	1.5	0.5	51	0.9	1.0	2.0	2.3
		D-3	19.9	43.1	5.2	18.8	5.1	2.7	6.9	0.9	4.1	1.8	2.8	0.5	3.9	1.0	117	2.0	0.9	2.3	2.2
		D-4	15.0	37.1	4.9	9.2	3.0	0.5	4.9	1.1	3.8	1.8	4.1	0.4	1.9	1.1	89	0.5	0.9	3.4	2.2
		D-5	13.1	29.3	4.1	11.1	2.4	1.7	4.1	0.5	3.2	2.1	4.1	0.5	2.0	0.9	79	2.4	0.9	2.3	1.9
	Downstream	D-6	14.0	38.0	4.9	11.0	4.1	2.0	7.0	1.0	4.9	3.1	3.0	1.0	1.9	0.5	96	1.7	1.0	4.0	1.8
		D-7	13.0	39.1	3.9	12.1	2.2	1.1	4.1	0.5	2.2	2.5	1.2	0.5	1.5	0.4	84	1.6	1.2	2.2	1.3
		D-8	18.1	42.8	4.2	7.9	3.9	0.5	4.5	1.0	5.0	2.8	3.0	0.5	2.5	0.5	97	0.5	1.1	3.6	3.4
		D-9	10.3	28.9	4.1	6.2	3.1	0.5	4.0	0.5	3.2	2.8	2.0	0.5	1.0	0.5	68	0.6	1.0	4.1	1.7
		D-10	15.2	35.0	3.2	11.0	4.9	0.5	4.2	0.5	2.1	1.1	2.0	0.5	1.5	0.5	82	0.5	1.1	2.4	1.4
		D-11	13.2	32.1	4.8	8.2	2.1	0.5	4.0	0.5	2.1	0.9	2.0	0.5	1.9	0.5	73	0.8	0.9	3.1	2.5
		D-12	23.2	55.2	7.3	19.0	3.0	1.1	6.0	1.1	3.0	1.0	3.0	0.5	2.2	0.5	126	1.1	0.9	2.0	1.2
		D-13	41.3	90.1	10.2	42.1	9.3	2.5	9.4	1.5	8.4	2.1	4.6	0.8	4.9	0.8	228	1.2	1.0	1.4	1.2
